# Vocational Training for General Practice

**Published:** 1985-07

**Authors:** Michael Lennard

**Affiliations:** General Practitioner, Bristol


### Vocational Training for General Practice— Achievements of the Last 15 Years

**Michael Lennard, M.B., Ch.B., F.R.C.G.P.**
General Practitioner, Bristol

In October 1968, this Journal published a paper entitled 'Vocational Training for General Practice' which was mainly concerned with the need for such training at that time, and the future developments necessary to make achievement a possibility.

In assessing the then current position, it was suggested that in its attempt to establish itself as a true academic discipline, General Practice had to demonstrate four criteria:

(i) a unique field of action;

(ii) a defined body of knowledge;

(iii) an active area of research;

(iv) an intellectually rigorous training.

The general feeling then was that the first two criteria were almost self-evident, and therefore 'achieved', the third was very slowly establishing itself, and that the fourth was the one that had been most neglected in Britain—and, indeed, elsewhere. Any forward planning to rectify this would, it was suggested, have to take into account certain clear trends which had become discernible in the previous decade:

(i) The growing concept of 'The Team' in General Practice.

(ii) Increasing interest in Preventive Medicine in its widest sense, including Health Education, pre-symptomatic diagnosis, the early recognition of disease.

(iii) The growth of our therapeutic range, and the resultant increase in the amount of illness treated outside hospital.

(iv) Developing awareness of the importance of the behavioural sciences.

(v) The possibility of a greater use of hospital beds by GP's; and their absolute need, at the very least, for full open access to diagnostic facilities.

(As an aside, it is odd to realise that most of these things are, now, taken for granted, then, they were thought to be worth listing in a discussion paper as growing points in the development of practice.)

The objective of Vocational Training was defined as the production of a young graduate who had made a positive choice of a Family Practice Career, who had followed a planned and defined course to train for it, and who was aware that education is a lifelong process. The facilities that would be required for this training were outlined: first, hospital appointments to follow, and complement the pre-registration year, and recognition by the specialists supervising those appointments of the aims of this phase of the GP's training: second, a GP component based on the Traineeship scheme set up in 1948, but with a much greater input from the trainers, and an increase in their numbers: third, a built-in academic component, and, perhaps, University involvement and supervision in the whole business.

Achievement of all this would depend on the right facilities being developed within the existing framework of medical care:

(i) Sufficient designated hospital posts, perhaps in groups and with rotating and combined appointments.

(ii) An adequate supply of trained GP teachers and traineeship posts.

(iii) Some incentive to proper training. Even if there were no frank financial incentive, there must at least be no disincentive (such as existed then for GP trainees compared with both specialist trainees and young intending GP's who did no formal training).

(iv) Some sort of overall authority governing this training, probably central, but working through the regions: these national and regional authorities it was suggested would need, full time or part time 'GP Post-graduate Advisers' as their working agents.

Finally, a plea was made for the University to take part in all this, so that it might be as involved with the future of General Practice as it was, inevitably, with the future of Specialism.

Now, more than fifteen years later, how far have we got? Is General Practice a recognised discipline? In 1968, there were only two fully established Departments of General Practice in the U.K., and some embryo ones: now there is a department or sub-department in every Medical School, with the notable exception of Bristol. GP Research has grown considerably; the Contraceptive Survey is the biggest piece of work, and one which shows the unique potential of such GP-based research. Training is established, and is now mandatory for the intending GP Principal; and if it is not yet as intellectually rigorous as it should be, at least the basis is there to build on.

Has practice developed as was predicted in 1968? Certainly the GP team is now well developed with District Nurse and Health Visitor and other attachments the rule rather than the exception. Prevention *is* now of greater concern: many more practices do their own Child Health work than formerly (though not yet enough have taken up the challenge) in line with the suggestions of both the Sheldon and the Court Reports: presymptomatic care is growing, e.g. cervical smears and follow-up are offered as a routine in many practices—but, again sadly, not all: local patients' groups are being developed in some practices for education and self-help purposes. The 'drug explosion' has profoundly affected practice, particularly perhaps in those groups of drugs used in emotional disorders, though this effect has not always been for the best. This particular facet of the drug field has linked up with, and has been a factor in promoting, interest in the behavioural sciences, and personal, social and group psychology have become fashionable talking-points. The desire for hospital beds remains a major preoccupation for some practitioners and in some areas; one doubts if it has grown, but we can be perfectly sure that genuine open access to hospital investigative facilities is now the universal rule; one has only to note the outcry if any facility is even temporarily withdrawn to feel sure the privilege will remain.

Finally, given that we have achieved a proper postgraduate training for all GP's, have we fulfilled the conditions considered necessary sixteen years ago?

(i) Hospital Appointments. The future GP spends a minimum of two years in selected and approved SHO posts, either by appointment to an official Vocational Training Scheme (VTS), or on a self constructed programme or rotation. There is not now a Hospital Group in the South-West Region that is not involved with a VTS: it seems likely that most such Hospital Groups would feel the draught quite badly if this supply of Hospital Officers disappeared.

(ii) Trainers. In 1970 there were some thirty appointed trainers in the South-West Region, not all of whom were 'active': now about one GP in ten is a trainer, viz. some one hundred and sixty in the region, and nearly all trainee posts are constantly active; in fact, there are more trainees in post in the South-West Region than there were trainees in the whole of England and Wales in 1965. All trainers have attended Trainer's Courses, all belong to a Trainer's Workshop—and most are reasonably regular attenders. Appointment, re-appointment, of a GP teacher is preceded by reasonably critical inspection and interview; re-appointment, after a maximum of five years, takes into account the comments of both local organisers and recent trainees.

(iii) Incentive. In the early years of the 'new look', actually about 1972 onwards, the Vocational Training Allowance (VTA) was sanctioned by the Government and this did something to promote the then voluntary arrangements: now that these are mandatory, this 'carrot' has become redundant.

(iv) Supervision. The control of standards for Vocational Training for GP was centred in 1972 on University Medical Postgraduate Committees: more or less at the same time in most regions. Regional Advisers in General Practice were appointed. National co-ordination came through the Councils for Postgraduate Medical Education in England and Wales and in Scotland: the Postgraduate Deans, through these Councils and through their own 'Conference', played a very important part in the developing scene: Regional Advisers, too, formed their own National 'Conference'. Eventually, after much discussion and indeed some delay, the Joint Committee for Postgraduate Training in General Practice was set up, and this body has the legal responsibility for overseeing the whole business; the registration of completed training; the inspection and approval of VTS; and the criteria for the appointment of trainers. In all this they work closely with and through the Regional Advisers.

So indeed the changes and achievements of fifteen years *are* considerable—but not complete. It seems likely that the next fifteen years will see changes no less considerable. Perhaps they might even include a Department of General Practice in the Medical Faculty of Bristol University! Roll on 2000.

### REFERENCE

LENNARD, M. B. (1968) Vocational Training for General Practice. *Bristol Med-Chi. J.* **83**, 23.

